# Why Are Viscosity and Nonlinearity Bound to Make an Impact in Clinical Elastographic Diagnosis?

**DOI:** 10.3390/s20082379

**Published:** 2020-04-22

**Authors:** Guillermo Rus, Inas H. Faris, Jorge Torres, Antonio Callejas, Juan Melchor

**Affiliations:** 1 Ultrasonics Group (TEP-959), Department of Structural Mechanics, University of Granada, 18071 Granada, Spain; grus@ugr.es (G.R.); inas@ugr.es (I.H.F.); acallejas@ugr.es (A.C.); 2 Biomechanics Group (TEC-12), Instituto de Investigación Biosanitaria, ibs.GRANADA, 18012 Granada, Spain; jmelchor@ugr.es; 3 Excellence Research Unit “ModelingNature” MNat UCE.PP2017.03, University of Granada, 18071 Granada, Spain; 4 Department of Statistics and Operations Research, University of Granada, 18071 Granada, Spain

**Keywords:** elastography, soft tissue, nonlinearity, viscoelasticity

## Abstract

The adoption of multiscale approaches by the biomechanical community has caused a major improvement in quality in the mechanical characterization of soft tissues. The recent developments in elastography techniques are enabling in vivo and non-invasive quantification of tissues’ mechanical properties. Elastic changes in a tissue are associated with a broad spectrum of pathologies, which stems from the tissue microstructure, histology and biochemistry. This knowledge is combined with research evidence to provide a powerful diagnostic range of highly prevalent pathologies, from birth and labor disorders (prematurity, induction failures, etc.), to solid tumors (e.g., prostate, cervix, breast, melanoma) and liver fibrosis, just to name a few. This review aims to elucidate the potential of viscous and nonlinear elastic parameters as conceivable diagnostic mechanical biomarkers. First, by providing an insight into the classic role of soft tissue microstructure in linear elasticity; secondly, by understanding how viscosity and nonlinearity could enhance the current diagnosis in elastography; and finally, by compounding preliminary investigations of those elastography parameters within different technologies. In conclusion, evidence of the diagnostic capability of elastic parameters beyond linear stiffness is gaining momentum as a result of the technological and imaging developments in the field of biomechanics.

## 1. Introduction

Elastography is a medical imaging modality intended to map the elastic properties of soft tissues for diagnostic purposes that has recently been undergoing heavy development. It combines an imaging principle, that is, either ultrasonic or magnetic resonance imaging (MRI), with algorithms to reconstruct the stiffness maps from the raw shear wave propagation data [1,2,3,4]. The references are more detailed on ultrasound elastography, given the variety of techniques and the author’s background, but the conclusions are fully applicable to magnetic resonance elastography (MRE). It follows that only dynamic or shear wave methods will be reviewed since strain elastography merely delivers relative deformability as the stress is unknown. However, in static ultrasonic methods, this current dependency of the stiffness on the probe pressure can become an opportunity instead of a drawback, since that dependency is caused by elastic nonlinearity, which is only quantifiable by dynamic techniques at this time. Further, the emerging field of elastography of viscous elastic parameters is finally gaining prominence. Therefore, beyond the current standard of elasticity maps, measuring the nonlinearity and viscosity might yield a more precise, pressure and operator-independent interpretation of the results, since for nonlinearity models, the dependence of the tangent stiffness modulus with deformation is correlated with operator-applied probe pressure, hence, decoupling operator dependency at the time a new biomarker is added. This proposed mechanical biomarkers, whose rationale is found in the tissue microstructure, and preliminary evidence, suggest a convincing diagnostic potential.

The purpose of this present work is not to address ultrasound elastography techniques in detail; there are several published works that deal with their differences and cut-off values, and the different systems available in the clinical market [2,5,6,7,8]. Instead, this article reviews the projected capabilities of viscous and nonlinear elastography parameters as clinical biomarkers from three perspectives: (1) the linear mechanics of soft tissue, focusing on the microstructure of the stroma, and therein mainly the fiber network organization; (2) how viscous and non-linear parameters are expected to be able to refine the diagnoses provided by classical elastography modalities; and (3) a spectrum of pathologies for which viscous and nonlinear elasticity quantification, conceived as mechanical biomarkers, has current or potential applications.

## 2. Mechanics of Soft Tissue

### 2.1. Soft Tissue Microstructure

**Figure 1:**
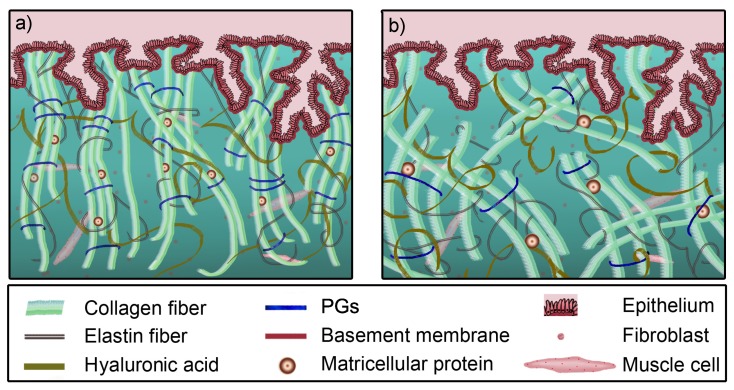
Histological illustration of the ECM remodeling as an example of cervical tissue during pregnancy. (**a**) The structure of the constituents in non-pregnant women. (**b**) The morphological evolution near the end of pregnancy. Quantitatively, there are increases in active cell and water contents, and crimping; and the diameter of collagen fibers increases, while PGs show a cyclic behavior. The legend at the bottom describes the symbol for each constituent; PGs: proteoglycans.

The application of imaging techniques based on the propagation of shear acoustic waves aims to become a benchmark in terms of medical diagnosis. Pathologies such as tumors and fibrosis involve changes in consistency, since the structural properties of these anomalies imply a stiffer area that reflects histological differences in the microstructure of the tissue [9]. For current technologies to be effective and reliable, a sufficiently broad range of variation in the mechanical properties of the tissue must occur. This response can be addressed at the biological microscale, where the most relevant information can be gathered [10,11,12]. At this scale, there are two fundamental components, the extracellular matrix (ECM) and active cells, with fibroblasts and smooth muscle cells being the most prominent of this second group. The integrity of the tissue is ensured by the ECM, with a composition that provides support for the structural functionality through the formation of a fibrous scaffold. The components are organized hierarchically down to the macromolecular level, according to the morphology and function of the tissue they form. The primary constituent of the ECM is a crosslinked network of collagen and elastin, which is embedded within a gelatinous matrix of proteoglycans (PGs). This matrix is responsible for resisting and transmitting mechanical loads and regulating the hydrostatic pressure and fluid flow [13]. For illustrative purposes, the reader is referred to Figure 1, where the remodeling process of cervical ECM during pregnancy is graphically described.

All the elements that compose the ECM have a load-bearing role in the mechanical response, emphasizing the importance of the content and distribution of collagen fibers in the shear modulus of the tissue. During the synthesis of collagen there is a process of hydroxylation that determines the crosslink formation, setting the adhesion of the new fibrils [14,15]. This is a critical step in the development of pathologies related to collagen [16], such as fibrosis-associated pathologies, as shown on foreskin cell cultures [17]. When several fibrils are adhered, they increase the crosslink density, creating a stiffer fiber, 1–20 μm in diameter [18], and completing the fibrillogenesis process [19,20,21,22,23]. Apart from the diameter, the morphology of the collagen is defined by the interfibrillar space and the crimping. The origin of this wavy structure comes from the subfibril formation, very sensitive to different homeostasis levels affected by biochemical factors. It can resist very low compressions due to this crimping, which in turn is responsible for the existence of internal shear [24,25]. The collagenous matrix varies greatly depending on the organ and its state. For instance, breast tissue has a collagen content of 5–10% [26,27], similarly to the liver [28,29]. The collagen content is higher in the cervix and prostate tissues: around 60% for the cervix [30,31,32], and similar content for the prostate can be inferred from qualitative analysis [33].

Elastin fibers are randomly distributed in the ECM, loosely interconnecting collagen fibers [34]. During their formation elastin fibers are prestressed, and once they are assembled they discharge stress, stretching and curling the attached collagen fibers [35,36]. They are used as a support in the mechanical response of collagen, operating as springs that recoil the structure to its initial state, allowing it to withstand repeated load cycles without reaching a plastic state [37]. They have a linear response up to 100% strain, with an average stiffness of 0.4 MPa depending on the tissue (two orders of magnitude lower than collagen) [38,39].

The gelatinous matrix is a ground material composed of water, proteins and PGs. PGs fill the spaces between the fibers in a perpendicular scattered network, conferring a supportive bending stiffness to collagen. At the same time, they contribute to resisting compression forces along with the interstitial fluid, balancing the fiber network [40]. PGs are composed of a core protein that covalently bonds with glycosaminoglycans (GAGs), thereby becoming a scaffold for the loose proteins of the ECM. Some of them can interlace their core with collagen, affecting the fibrillogenesis [41] and providing lateral stability [42,43]. GAGs are polysaccharide macromolecules with high electrical charges; among them, there is a particular GAG called hyaluronic acid, which is able to imbibe the surrounding elements. This is a hydrophilic process that attracts water, generating osmotic pressure, turning these components into a dampener against compression [44].

### 2.2. Linear Elasticity

**Figure 2:**
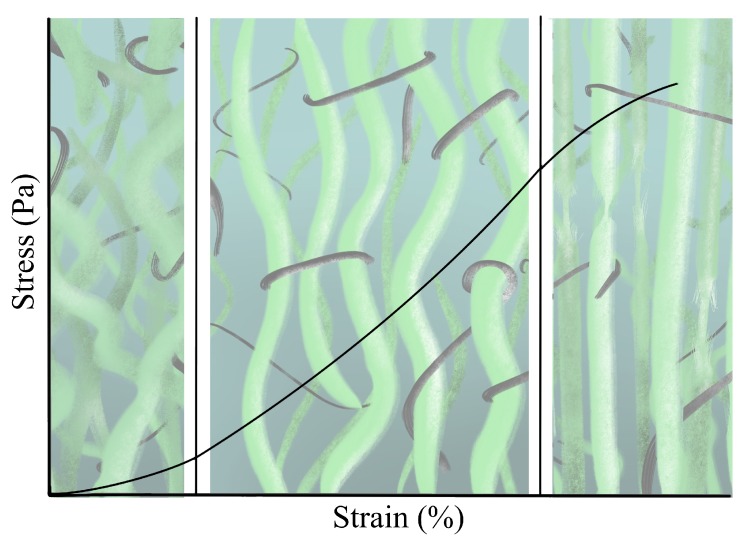
Stress–strain curve in soft tissues. The relationship is divided into three regions; namely, the toe, the nearly linear, and the failure regions: in the first, elastin fibers absorb most of the deformation and collagen forms a loose network that offers little resistance—primarily nonlinear behavior; in the second, collagen fibers line up and start to work under severe stress (nearly linear); and in the final region, the maximum capacity is reached. Color codes for the fibers are green for collagen and black for elastin.

The heterogeneous combination of the ECM components exhibits directional anisotropy, which is mainly attributed to variations in the morphology of the crosslinked fiber network [45]. Consequently, the stress at a point does not depend only on the gradient of deformation but also on the orientation, connection and distribution of its components. At the same time, the fiber network displays a nonlinear stress–strain relationship due to complex interactions that vary from point to point. The action of collagen and elastin can be lumped together, showing a stress–strain behavior divided into three regions (see Figure 2) [46]: (i) In the absence of load, collagen fibers are in their natural state of formation, wavy and loose. Due to its symmetrical organization, its behavior is frequently modeled as approximately isotropic. For strains lower than 2%, collagen offers little resistance, originated by fiber bending; thus, it is considered that elastin absorbs most of the energy, acting as a spring. This is the area with normal physiological activities called the toe region, showing nonlinear effects [47]. (ii) The progressive increase in deformation will disrupt the fibers that begin to line up in the direction of the load increasing the stiffness; this in turn, means that crosslinks are stressed and interfibrillar sliding is induced; the stress–strain relationship is approximately linear. (iii) At around 30% strain, depending on the tissue, crimping disappears and the fibers are arranged in parallel; the tissue reaches its highest stiffness [48,49,50]. Beyond these values, crosslinks and fibers begin to break, leaving severe damage to the tissue.

Current imaging technologies are gaining prominence because they are based on the propagation of shear waves, which is directly proportional to the shear modulus, a very sensitive parameter to the microstructure of the material being examined [51]. Whether through biochemical modulations or the presence of a disease, the integrity of the tissue changes, which might be quantifiable with enough contrast for a clinical diagnosis; for that purpose a range of scores has been proposed [8]. However, it is difficult to maintain a standard, as the review article of Sigrist et al. notes [2], because the characterization of the shear modulus in the same tissue is variable. The variability in the commercial equipment methodologies and the existence of mechanisms of contrast make achieving standardization unfeasible. Another factor comes from the physical nature of shear waves; the displacement generated is characterized by being usually oriented perpendicularly to their propagation. However, the waves do not propagate with the fibrous matrix direction necessarily. This is dependent on the interaction of the wavelength relative to the interrogated fibrous matrix; therefore, its inherent anisotropy defines the examined direction. The dependence on tissue anisotropy, albeit interesting, is outside the scope of this work; for further information the reader is referred to [45]. Additionally, when the viscoelastic nature of tissues is considered, their mechanical response is time and frequency-dependent. Finally, the microscopic dimensions of the ECM components concerning the exciter wavelength must be taken into account. The key is to find a trade-off in the excitation frequency between a small enough wavelength that is able to interrogate internal components of the target tissue and a distance to the source of excitation that reduces wave attenuation [52,53,54].

The next step is to introduce the mathematical basis for soft tissue biomechanics, in this case, from the perspective of the continuum making two simplifications. The first is incompressibility, which stems from the high water content that does not allow the tissue to alter its volume under deformation; thus, the Poisson’s ratio is considered close to 0.5. The second simplification is isotropy, since the anisotropy of the tissue increases the difficulty of formulating robust constitutive relationships. In most soft tissues, these simplifications have enabled researchers to work with more manageable problems, enabling progress in the understanding of the mechanics of soft tissues.

The total stress ($\sigma_{ij}$) and strain ($\varepsilon_{ij}$) can be deconstructed into two linear parts that naturally decompose the basic constituents of soft tissue [55,56]. On one hand, the volumetric, spherical or hydrostatic part is associated with the ground substance, mainly fluid, which provides no significant stiffness against shear deformations but is highly incompressible. On the other hand, shear stiffness is provided by the stroma, which governs the deviatoric components, the fiber and protein structure of the ECM.

(1) $$\sigma_{ij}=-p\delta_{ij}+\tau_{ij}\;p=-1/3\sigma_{kk}$$

(2) $$\varepsilon_{ij}=-v\delta_{ij}+d_{ij}\;v=-1/3\varepsilon_{kk}$$

where $\delta_{ij}$ is the delta of Kronecker, *p* is the hydrostatic pressure, *v* is the volumetric strain, $\tau_{ij}$ is the deviatoric stress tensor and $d_{ij}$ is the deviatoric strain tensor. The previous relations can be combined to derive a constitutive relation, which is linear at first approximation and is similarly divided into volumetric and deviatoric components.

(3) $$\sigma_{ij}=\lambda \delta_{ij}\varepsilon_{kk}+2\mu \varepsilon_{ij}$$

where λ and μ are known as the Lamé constants, which characterize the elastic behavior of the material and must be obtained experimentally. The constant λ has no direct physical meaning; nevertheless, it is often associated with the bulk modulus K=λ+2/3μ, which describes the response in volume change under volumetric pressure. Since the compressibility of soft tissues tends toward that of water, which is orders of magnitudes higher than shear stiffness provided by the stroma, a good approximation is K≈λ. As for the constant μ, it is usually called shear modulus and represents the resistance to shear deformation and can be written in terms of elasticity modulus and Poisson’s ratio μ=E/(2(1+ν)) [57]. The volumetric and deviatoric decomposition naturally splits the former linear constitutive relationship into

(4) $$p=3Kv\;\tau_{ij}=2\mu d_{ij}$$

Nevertheless, these parameters and assumptions do not provide a full representation of the behavior of soft tissues; they are limited to low levels of strain, such as image-guided interventions. New methodologies to interrogate other mechanical properties, such as shear viscosity and shear nonlinearity, are now appearing. The viscoelasticity of soft tissues implies the search for high order models that characterize the dispersion associated with shear wave propagation. As input, some studies have used the shear wave group velocity, which approximates as a series of derivative orders [58,59]. Likewise, taking advantage of the acoustoelasticity phenomenon, wherein the shear wave velocity is altered when a stress is applied due to wave propagation, new parameters become measurable [60,61,62]. For the specific case of nonlinear values, several theoretical methodologies have been proposed, and some experimental results have been obtained through acoustoelasticity, high amplitude shear wave propagation and nonlinear shear wave interaction [63]. The extracted information refers to the structure and functionality of the tissue, allowing one to identify conditions that elasticity alone is not able to capture so that the diagnosis is refined.

### 2.3. Viscoelasticity

From the mechanical viewpoint, two phenomena contribute to the time-dependent or rheological behavior of soft tissues: viscoelasticity and poroelasticity [64]. Although both viscosity and porosity contribute additively to the same phase lag between stress and strain dynamics, they are commonly quantified as an unique value called viscosity within the elastography community. However, viscoelasticity and poroelasticity stem from fundamentally different origins and are only separable playing with space and time scales. In other words, at large-size scales, tissues are viscoelastic in the short-time period and poroelastic in the long-time period, whereas the small-size scales, tissues are poroelastic in the short-time period and viscoelastic in the long-time period [65], which is clinically intractable given the limited region and frequency ranges. For this reason, it might be appropriate to rename viscoelastic elastography to rheological or dynamic elastography.

Soft tissues are generally assumed to be decomposed into their porous solid phases and their fluid phases [66]. The high fluid content in tissues is combined with the poroelastic structure of the ECM to allow motion between components under load, creating a time delay in the strain and triggering the viscoelastic response [67]. This biphasic nature implies a phase lag between the stress and strain associated with a relaxation time, or in the case of oscillatory mechanical tests, a phase angle. Then it would be advisable to start considering time-dependent effects, since the strain response to load and unload conditions is a function of time, often called the velocity of deformation. During the loading cycle there is dissipation of energy, reflecting the existence of hysteretic effects. At the same time, the strain evolution is slowed to allow the viscous flow to settle. Thus, the duration and rate of loading define the dynamics of the tissue strain. Without this characteristic, the stress during physiological activities would be harmful to the active structure [68].

One of the key features of viscoelastic tissues comes from the physics of wave propagation, where the dispersion is defined as a compound expression of the poroelastic and microstructural media governed by the complex fibrous multiscale microstructure of the stroma [69,70,71,72]. It is also known that the amplitude and intensity of waves decays proportionally to the distance traveled. Additionally, in a highly viscous environment, where the microvasculature and hemodynamics play an important role, it is observed that wave phase velocity changes with frequency, and wave amplitude is affected by geometric factors, such as boundary conditions and the sizes of scattering particles, similar or smaller than the wavelength [73]. Another important point is that the frequency-dependent behavior complicates the comparison of different technologies, since each author chooses a suitable range [6]. Neglecting the viscous part introduces bias for the estimation of elasticity, since the effect of wave dispersion is ignored.

The possibility of explaining these mechanical parameters by the internal structure and function of the tissue seems to be the key to improving the specificity of a pathology diagnosis. Collagen by itself exhibits viscoelastic behavior, attributed to fiber and fibril sliding and the crosslinking density; however, due to its short time of relaxation, it seems that the global response is dominated by non-collagenous components [74]. Elastin has been found to contribute to stress relaxation, since when it was removed in arteries, the relaxation time dropped significantly [75]. Nonetheless, PGs are considered to be the main viscous constituents, via embedding the collagen fibers and creating a lubricating effect. Their hydrophilia generates hydrostatic pressure, which, coupled with HA [76] and its large molecular size, entails water attraction, filling the porous matrix [77]. The roles of PGs and HA have been reviewed in tumor biology [78] and in inflammatory processes [79]. They are capable of acting as signaling pathways, interacting with diverse receptors, which affect the ultrastructure of the ECM that is transformed during inflammatory and neoplastic diseases [80]. In the case of pregnancy, as the time of delivery approaches, an inflammatory process is triggered, during which the proportion of PGs to collagen increases; therefore, higher viscosity is expected [81,82,83]. As for fibrosis disorders, there is an increased deposition of ECM constituents, especially collagen, accompanied by PGs and HA that help in cell signaling and proliferation [84]. A better understanding of these proteins and their relationship with viscosity might allow for the development of concrete diagnostic and therapeutic strategies.

Similarly, higher smooth muscle cell (SMC) tone in the carotid wall has been linked to higher viscosity [85]. For its part, it has been seen that there is an increase in SMC in the internal walls of the cervix as delivery approaches, and at the time of induction it became the most sensitive part [86]. In the liver, the development of fibrosis has been accompanied by an increase of SMC actin [87]. Investigations about the arterial viscoelasticity linked it to wall pressure [88]. From the perspective of tumors, there are changes at the cellular level which promote different reactions of the stroma. In breasts, the viscosity of lesions has been studied in order to discriminate the nature of the masses [89,90,91,92]. Higher viscosity was registered compared to healthy tissue and different ranges allowed researchers to distinguish between benign and malignant lesions.

Thus far, most studies have ignored this behavior, relying only on approaches based on linear elasticity simplifications. Although this has enabled progress to be made in quantitative imaging techniques, diagnoses sometimes fail because they do not deal with all the information. [93]. To reduce false-negative and false-positive results and to better understand pathological changes in soft tissues, extended dynamic mechanical parameters such as viscosity need to be investigated [94] and eventually be used as new diagnostic biomarkers. Ex vivo studies evidence the predictive relationship between viscosity and pathology; for instance, the marked Ex vivo neuronal demyelination with development of apparent vacuoles associated with a loss of interneuronal connections and thus with a reduction of matrix dimensionality, causing an observed alteration of viscosity [72,95,96,97]. The collected data from either traditional testing methods (creep and relaxation tests) or state-of-the-art imaging combined with the current computational power are allowing for the retrieval of viscous parameters from empirical or computational models. Table 1 presents a preview of the experimental evidence from which viscosity parameters have been estimated with different methods, along with applications to soft tissues whose results are described later in the manuscript.

It is important to note that if tissues are precompressed when they are examined, the estimation of parameters will be biased, as the time-dependency of the response is relevant. Changes over time due to mechanical stimulation are attributed to rapid alterations in cellular activity, mainly the synthesis and modification of components of the ECM (collagen and proteinases) [112]. To avoid this situation, preconditioning protocols should be proposed whenever the specimen studied allows it, so that a stabilization in the response is achieved [113]. With the aim of capturing this material behavior, the most popular approach considers soft tissues as uniphase solids and their response to external loads or deformation is represented as a lumped relationship. This method uses linear viscoelastic models that generally include a solid-related characteristic (e.g., spring) and a viscous fluid element (e.g., dashpot). To name a few, Maxwell, Kelvin–Voigt (KV) and Zener viscoelastic models provide information on how the different scales are linked to each other [98,111]. However, in order to fit a model when the soft tissue shows several characteristic times, generalized linear viscoelastic models are used, such as generalized Maxwell or KV models [114,115]. When large strains are expected, these linear models are not suitable; thus, the proposed Fung’s quasilinear viscoelastic model is frequently adopted [116].

One of the models in the literature most used to fit the parameters is the KV model, due to its simplicity [117]. Other models have been explored, such as Maxwell; fractional derivative versions of the above; and combined models, such as the springpot model [118]. The KV formulation in terms of the stress tensor (Equation (1)), assuming constitutive and viscous linearity have been derived with the aim of simplifying equations [119]. Following the references found in the literature [120,121,122],

(5) $$\begin{array}{c} p=3Kv+3\eta^{v}\dot{v}\\ \tau_{ij}=2\mu d_{ij}+2\eta {\dot{d}}_{ij} \end{array}$$

where *K* is the compressional modulus; η and $\eta^{v}$ are the shear and volumetric viscosities, respectively; and $\dot{v}$ and ${\dot{d}}_{ij}$ are the derivate of the volumetric and deviatoric strains, respectively.

Assuming incompressibility, only deviatoric components ($\tau_{ij},\;p=\nu =0$) are considered. According to the schematic representation of the KV model, the total stress is the sum of the elastic and viscous terms,

(6) $$\sigma_{ij}=\tau_{ij}=2\mu d_{ij}+2\eta {\dot{d}}_{ij}=2\mu \epsilon_{ij}+2\eta {\dot{\epsilon }}_{ij}$$

Following the same steps as in the Kelvin–Voigt model, the implementation of the Maxwell model stems from the strain tensor of Equation (2). For the same reasons stated for the KV case ($d_{ij},\;p=\nu =0$), exclusively deviatoric components are considered. Only elastic and viscous components of the deviatoric term of the strain tensor are adopted,

(7) $$d_{ij}=\tau_{ij}/2\mu ,\;\;\;{\dot{d}}_{ij}=\tau_{ij}/2\eta$$

The constitutive equation for this model is obtained by adding the elastic and viscous terms by,

(8) $${\dot{d}}_{ij}={\dot{\tau }}_{ij}/2\mu +\tau_{ij}/2\eta$$

All this evidence suggests that the viscous phase may become a biomarker for the characterization of microstructural changes [123,124,125,126,127]. Table 2 shows some indications of the current status of this parameter in terms of limitations and characteristics that have been specified for some ultrasound elastography methods. Phase-sensitive imaging techniques might become a monitoring tool for early diagnose, able to keep track of quick dynamic changes in the tissue, before significant or unclear changes in elasticity and also reducing the number of unnecessary biopsies [1,128].

### 2.4. Nonlinearity

One of the main hypotheses about the pathology-mediated origin of nonlinearity changes is based on the nonlinear character of the strain response. The organization of collagen fibers and elastin, as well as their amounts, combined with the synthesis and degradation processes that are experienced due to growth and remodeling enhance the nonlinear behavior [129,130]. Additionally, the stress–strain behavior of the stroma is nonlinear between tension and compression, with a stiffer response and reduced extensibility in tension, and a more compliant response in compression [131,132].

Several experimental studies, including the recent study of Aristizabal et al. [94], estimated the nonlinear shear modulus in Ex vivo samples. Particularly, that paper was about Ex vivo kidneys diagnosing end-stage renal disease, for which a better contrast in the diagnosis was shown. Based on the principle of acoustoelasticity, the feasibility of obtaining nonlinear parameters through changes in the deformation and its consequent interaction with the propagated wave is proven. The application of a deformation and the use of radio frequency ultrasonic signals to quantify it, was the work of Goenezen et al. [133]; they obtained spatial maps of nonlinear elastic parameters in patients with malignant and benign tumors. Their conclusions highlight a greater magnitude in the case of malignant tumors. In the context of preterm birth assessment, Myers et al. [131] investigated the interaction between mechanical and chemical properties of several cervical samples from different human hysterectomy specimens: non-pregnant patients with previous vaginal deliveries; non-pregnant patients with no previous vaginal deliveries; and pregnant patients at the time of cesarean section. The samples were tested under confined compression, unconfined compression and tension. Results indicated that the cervical stroma has a nonlinear behavior that could be explained with an accurate multi-scale model.

The significant hyperelasticity that soft tissues exhibit can manifest itself as quantifiable shear wave harmonic generation (via ultrasonic shear elastography); the stored strain energy is variable with the fiber orientation. Taking this opportunity, an efficient application of nonlinear or hyperelastic constitutive equations for either finite element analysis or experimental analysis requires the derivation of a strain energy function to consider an adequate stress–strain relationship. A diversity of approaches to nonlinear mechanics have been developed since Landau and Murnaghan [134,135], which are particularly well-suited for nonlinear wave modeling; then came the recent proposals lead by Ogden, Mooney-Rivlin, Yeoh and Fung [136,137] which cover adjustment theories based on modeling of physiological mechanics [138].

These behaviors can be modeled from the perspective of the continuum, making the assumption of Landau third and fourth-order elastic constants (TOEC and FOEC),

(9) $$S_{ij}=\lambda \delta_{ij}\varepsilon_{kk}+2\mu \varepsilon_{ij}+A\varepsilon_{ij}^{2}+2B\varepsilon_{ij}\varepsilon_{kk}+C{\left(\varepsilon_{kk}\right)}^{2}+h.o.t.$$

limited to the third order, where A,B and C are the TOEC, and $\delta_{ij}$ is the delta of Kronecker, and where $S_{ij}$ is the second Piola–Kirchoff Stress tensor [139]. The simplification of nonlinear strain energy function in the case of incompressible tissues, and extended to fourth order, was derived by Hamilton [140], and it is considered as the most representative.

(10) $$S_{ij}=2\mu \varepsilon_{ij}+A\varepsilon_{ij}^{2}+4D\left(\mathrm{tr}\varepsilon_{ij}^{2}\right)\varepsilon_{ij}$$

Experimentally, nonlinear parameters can either be estimated by measuring the change of apparent speed of shear wave propagation after a precompression [141], or by quantifying the cumulative harmonic generation during the propagation of shear waves across nonlinear tissue [142]. The nonlinear shear wave equation depending on TOEC and FOEC in the soft solid isotropic state was derived by Hamilton and Zabolotskaya [140]. Then, through a strain energy function they were able to separate the compressional and shear parts. In that approach, nonlinear propagation depends only on three elastic constants of the first (linear), the third and the fourth-order (nonlinear). Therefore, the generation of harmonics in soft tissue and biomaterials is likely to be studied under this prism. However, it is also possible to describe a theoretical model of shear waves propagating in soft biological tissue induced remotely by the nonlinear radiation force of the focused ultrasound. The spatial and temporal profiles of the shear displacement confirm the results of the mathematical modeling previously described. The experimental procedures based on acustoelasticity techniques are also performed to obtain the nonlinear coefficients of the Burgers Equation by describing the behavior of tissue [63]. For example, another experience in this line of research is the use of MRE by visualizing the nonlinear propagation of shear waves providing valuable information about the nonlinear mechanical behavior of the soft tissue [143]. From this procedure, it is shown that both odd and even higher harmonics are processed, with their amplitudes depending on the actuator details, the image geometry and the nonlinear properties of the tissue. With an adequate analysis of the displacement, it is possible to derive the harmonics that arise from the nonlinear soft tissue response. They have been extracted, for example, in phantoms at 600 and 750 Hz. Thus, if strain energy is modulated, it is feasible to determine the nonlinear biomechanical properties of the tissue [51]. The second approach has been proposed in combination with torsional wave elastography, described later [144,145] following Landau’s theory [134] and its adaptation for quasi-incompressible media coupled with multiscale hyperelastic models [146,147]. The formulations of the nonlinear torsional wave propagation on a hyperelastic material should be taken into account in cylindrical coordinates characterized by strain energy functions [148,149].

Analogously, it is also possible to accurately and quantitatively recover the local Landau A parameter. The characterization of the shear nonlinearity of soft tissues by applying the acoustoelasticity techniques in quasi-fluids could be correlated to the ultrasonic shear wave speed [150]. But these theories should be tackled by more profound studies due to the dispersion and variability of the outputs. It is also possible to deduce the nonlinear coefficients in the modified Burgers model using the numerical simulation from the quadratic wave equation rewritten in its nondimensional form [63]. It has been introduced to calculate nonlinear parameters of hydrogels and in Ex vivo porcine kidneys [94], but the cubic orders are valid under a relation that should be verified in some cases depending on acoustic nonlinearity [140].

In summary, since shear waves are believed to be far more sensitive to tissue classification than standard compressional waves, but they are complicated to quantify, some experimental observations may tangentially suggest that nonlinear mechanical properties may be a key signature withh which to quantify and classify soft tissue behavior [151,152,153,154,155,156]. The advantages and disadvantages of the current scene of nonlinearity in biomechanics are summarized in the Table 3. Therefore, the focus on developing nonlinear models in the clinical field will provide a better understanding of soft tissue biomechanics alongside new diagnostic biomarkers. Techniques such as shear wave elastography and torsional waves are postulated to be crucial tools, sensitive to the measurements of these nonlinear parameters, provided a consistent and efficient complete formulation is established.

## 3. Clinical Applications

Since the 80s, elastography has gradually become a widely applied medical imaging technique [157]. The different techniques of elastography are based on the assumption that soft tissues are deformed more than rigid tissues, and that these differences can be quantified [158]. However, this conventional perspective is undergoing a change of scenery; recently, emphasis has been placed on the complex structures that soft tissues exhibit, deeming not only elastic but strongly nonlinear hyperelastic, viscoelastic and poroelastic behavior important. Linear elastic models have been used extensively to characterize soft tissues by the biomechanics community, though it is known that this simplification in the characterization provides incomplete information in their results. Additional biomarkers, such as viscosity and nonlinearity, are herein proposed as hypotheses to enable new diagnostic standards in a broad spectrum of pathologies. In the following subsections, because of the prevalence of the diseases from which they suffer, the focus is on prostate, breast, liver and labor disorders, not to mention that the conclusions could be extended to solid tumors, atherosclerosis and osteoarticular syndromes, to name a few.

### 3.1. Prostate

Prostate cancer is the second most common cancer in men worldwide (almost 1.3 million diagnoses) and the fifth leading cause of cancer death among men (350,000 deaths worldwide) [159]. Furthermore, the increase in longevity and awareness of the disease is leading to more men requesting screening, which in turn will dramatically increase the number of patients diagnosed [160]. Barr et al. [161] provided an extensive study of guidelines and recommendations on the clinical use of ultrasound elastography on the prostate.

Ex vivo and in vivo results have demonstrated that acoustic radiation force impulse (ARFI) can be applied to visualize internal structures and to detect suspicious lesions in the prostate [162,163]. Among all the elastography techniques for prostate cancer detection that provide quantitative elasticity results at present, transrectal SWE (TR-SWE) by Aixplorer^®^ (SuperSonic Imagine, Aix-en-Provence, France) is the most prolific in terms of the number of publications. Recent in vivo studies on prostate cancer diagnosis using TR-SWE presented auspicious results [151,164]. However, TR-SWE has some drawbacks [165]: the pressure artifacts induced by the transducer, as the end-fire design of the probe requires bending to image mid prostate and apex; the slow frame rate, i.e., one image per second; the limited size of the ROI, since only half of the prostate is covered; the delay in stabilizing the signals at each acquisition plane; and the signal attenuation in large prostates was making the evaluation of the anterior transitional zone of said prostates difficult or impossible [166]. Most of the quantitative elastography results of tissue elasticity of the prostate have been achieved by using TR-SWE in different states of in vivo prostatic tissue [151,166,167,168,169,170]. The frequency range is expected to be between 50 and 450 Hz according to other TR-SWE publications [171]. By analyzing these results, differentiation between benign and malignant tissue in terms of stiffness is not a trivial matter, since ranges of values overlap. In order to discriminate in vivo malignant tissues from benign tissues using TR-SWE, Correas et al. [164] and Barr et al. [151] proposed Young’s modulus thresholds of 35 and 37 kPa respectively. According to their conclusions, these thresholds provided additional criteria for prostate cancer detection and biopsy guidance and enabled a substantial reduction in the number of biopsies.

The application of point shear wave elastography (pSWE) allowed Zhai et al. [172] to reconstruct the shear modulus values from excised human prostates with different pathologies. The limitation of the work was the low spatial resolution, which may cause variations in the reconstructed shear modulus. Another in vivo study by Zheng et al. stated that pSWE could effectively measure the stiffness of prostate nodular lesions between prostate cancer and benign prostatic hyperplasia [173]. Even so, the authors specified that the limited detected depth and the fixed box dimensions of the target region of interest (ROI) could hamper the broader application of pSWE technology.

**Figure 3:**
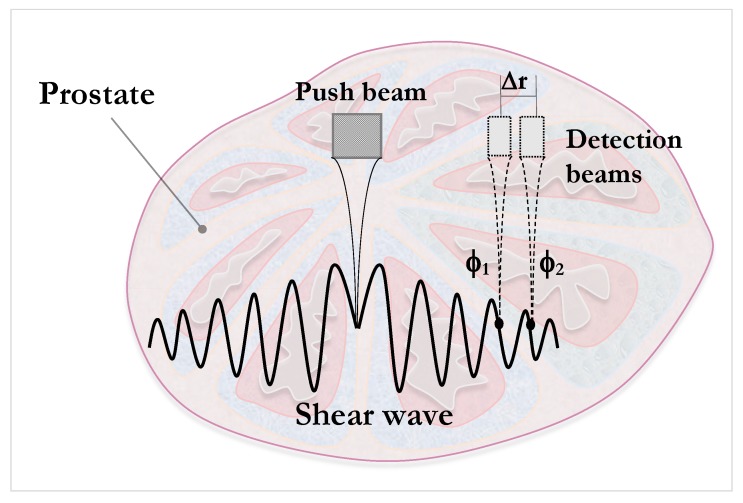
Illustration of shearwave dispersion ultrasound vibrometry (SDUV) principle. A harmonic shear wave is produced by a push beam; the propagation is monitored by separated detection beams at two positions. The shear wave speed is reconstructed from its phase $\phi_{1}$, $\phi_{2}$, separated a distance Δr.

As for the viscoelastic characterization of human prostatic tissue, few studies based on ultrasound elastography have addressed the issue. Shear wave dispersion ultrasound vibrometry (SDUV), one of the few techniques that has been used in the prostate, consists of monitoring the propagation of the shear wave by a separate ultrasound detector and reconstruction of the wave speed from two different phases [98] (refer to Figure 3 for an illustrative example of the principle). The in vitro study of Mitri et al. [101] used a KV model aimed at the characterization of the mechanical shear parameter for frequencies between 50 and 400 kHz. They obtained shear elastic modulus values of 1.31–12.81 kPa and viscosity values between 1.10 and 6.82 Pa.s. These data proved the viscoelastic nature of the properties of prostatic tissue.

Two other studies used a Kelvin–Voigt fractional derivative (KVFD) constitutive law, a more generalized case of the KV model, for measuring the variation of the complex Young’s modulus $E^{*}$ between normal and cancerous prostatic tissue [102,174]. In the first in vitro study, Zhang et al. [102] extracted the complex Young’s modulus by fitting data from a dynamic mechanical analysis (DMA) test to a KVFD model. In Table 4 the viscosity parameter and the order of the fractional derivative associated with the KVFD Young’s modulus is presented. In the second Ex vivo study, Hoyt et al. [174], made a comparative study between crawling wave spectroscopy and the same DMA test used in the first study for two samples of human excised prostate. Results showed relative similarities between techniques with errors below 12%. In any case, the sample sizes were too small to be statistically significant in both studies.

In the field of MRE some studies have addressed the generation of shear waves using transurethral devices. Chopra et al. [175] designed a transurethral actuator to produce shear waves in the prostate with adequate propagation at a reasonable frequency. A canine experiment demonstrated the feasibility of transurethral MRE in vivo. Shear waves have a penetration depth of 3–5 cm, as opposed to 15 cm for an external driver, allowing high spatial resolution. An alternative to the invasive transurethral driver was subsequently proposed by Arani et al. [176]. The driver was tested in prostate-mimicking gelatin phantoms to explore the imaging parameters of transurethral MRE and to determine whether they encompass the requirements for prostate cancer localization. A more recent study carried out by Reiter et al. [177] investigated the limitations present in MRI, such as interobserver variability and low specificity. For this purpose, fourteen fresh prostate specimens from men were examined. A piezoelectric actuator induced radially converging shear waves in the sample. The results of the work suggested that prostate MRE has the potential to improve the diagnostic performance of multiparametric MRI. An in vivo study carried out by Li et al. [108] showed that MRE could be used to distinguish between prostate cancer and benign prostatic disease in terms of shear viscosity. The study included 18 patients (eight with prostate cancer, 10 with prostatitis). The mean shear viscosity was significantly higher in prostate cancer (6.56 ± 0.99 Pa.s) than in benign prostatitis (2.13 ± 0.21 Pa.s).

Further experimental characterization studies of prostatic tissue are required to accurately model the real viscoelastic behavior of the prostatic tissue in all its conditions. As far as we know, no clinical studies taking into account the effect of the nonlinearity of prostate tissues have been reported.

### 3.2. Breast

The International Agency for Research on Cancer concluded in 2018 [159] that breast cancer is the most commonly diagnosed (over 2 million cases) and leading cause of cancer death (over 600,000 cases worldwide) among females. In the last few decades, several studies have compared the efficacy of the diagnosis of mammary elastography versus conventional ultrasound in the evaluation of different breast lesions. Ultrasound evaluation is established through the BIRADS classification [178], while the elastographic assessment is based on building a pattern between stress and size relationships [179,180]. Despite these efforts, it remains a significant healthcare problem, and what is more, countries in transition are experiencing a rise in their rates [181].

The representative clinical cases whose applications are relevant to include are benign lesions, malignant lesions and lymphatic and metastatic lesions [182]. The anatomy of the breast has allowed several elastography-based studies to be performed for the characterization and detection of masses. An extensive work of the World Federation of Ultrasound in Medicine and Biology (WFUMB) dealing with the guidelines and recommendations for clinical use of ultrasound elastography on the breast could be consulted for further information about elastography systems and their cut off values [183]. However, no clear consensus has been reached as to what measure of the shear modulus should be used and what ROI is the most appropriate for the estimation of elasticity. What is clear is that malignant lesions show a larger shear modulus than benign [92,184,185,186,187,188,189,190]. Still, several forms of misdiagnosis have been considered. The size of the lesion combined with a high density of the tissue could complicate the detection [191]. If benign and malignant lesions overlap, the power of the elasticity estimation is reduced [171,192]. Another issue comes from the effect of calcification: the surrounding zones are hardened, making the elasticity estimation higher. If the ROI selected matches this area, a misdiagnosis may be expected [193].

**Figure 4:**
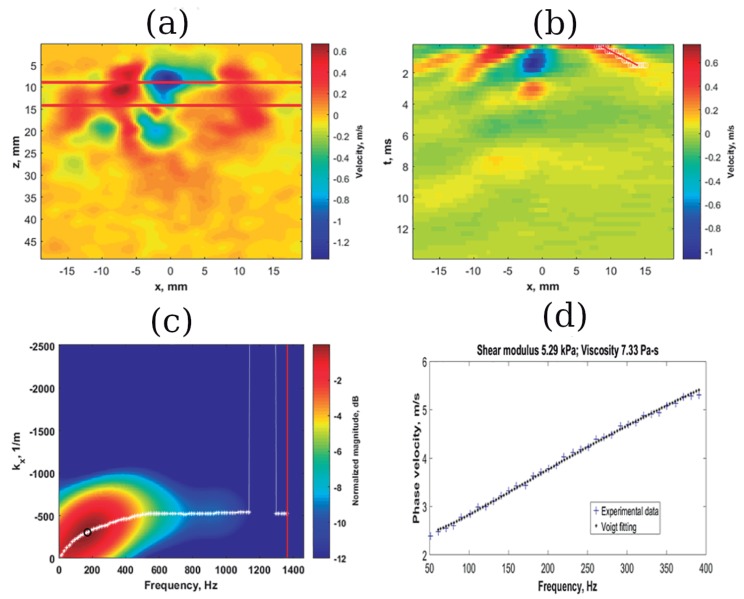
An illustrative process for the estimation of the viscosity parameter of a malignant mass. (**a**,**b**) Maps of particle velocity; (**c**) a k-space map displaying the phase velocity with the energy of each frequency; (**d**) the final result as a dispersion curve, based on the phase velocity, which is fitted using a Voigt model for estimation of viscoelastic parameters. Source: PLoS ONE, modified from 2018 Kumar et al. [89].

In contrast, viscosity is emerging as a better indicator, especially for tumor differentiation [194]. The first studies in using this parameter for in vivo diagnosis were attempted by Qiu et al. [195]. They compared the retardation times of benign and malignant lesions. The time for the benign state was clearly larger than the malignant. This was justified because malignant tumors increase their collagen and crosslinking densities, while there is a reduction of proteoglycans that declines the lubricating effect. Benign lesions are dominated by the fluid viscous phase of the tissue, hypothesized in part to be the lubricated motion of collagen. Those results are opposed to the studies on the quantification of the shear viscosity summarized in Table 5. Sinkus et al. performed two in vivo studies using MRE [103] and transversely isotropic models [104]. The idea behind the use of models with transverse waves is to remove the contribution of compressional spurious waves in order to reconstruct viscoelastic parameters. The SDUV technique has also been used in combination with viscoelastic models [89] (refer to Figure 4 for the reconstruction process). Another recent study on in vivo tissue applied the data from the creep test to a first order KV model fit, where the retardation time allowed them to distinguish between benign, malignant and healthy tissue [196]. These techniques are not feasible to compare, since, as previously stated, soft tissues are frequency-dependent and each author uses a different range of frequencies. The common finding that emerged was that shear viscosity was higher in all malignant states, and despite the great dispersion showed, these masses were heterogeneous in terms of their viscosity values. Additionally, the studies inferred that the maximum values were well correlated with malignant diagnosis in MR mammographies, encouraging further exploration.

Bernal et al. [141] focused on the detection of early breast cancer in vivo by nonlinear quantification. In their study they implemented a technique that combines shear wave elastography with a prestress that modifies the shear wave speed due to the Landau-type elastic nonlinearity, to measure the nonlinear shear modulus. The mean values of the nonlinear parameter A were −95 kPa for healthy tissue, −619 kPa for benign lesions, and −806 kPa for malignant lesions, a considerable variability that show signs of its utility.

These techniques suggest a promising scenario, but the recent expansion of elastography among all device designers and manufacturers has led to a dizzying increase in the number of tests whose results call for consistency improvements [197]. Despite this, it has been exhibited that both linear and nonlinear elastography, possibly together, promise better sensitivity and specificity with which to characterize benign and malignant mammary lesions [198].

### 3.3. Liver

Over two million people are estimated to die every year due to chronic liver diseases: one million due to complications of cirrhosis and the rest due to viral hepatitis and hepatocellular carcinoma [199]. These diseases remain a burdening health problem [200] that demands better mechanisms for prevention, correct detection and treatment [201]. Different organizations have published a quite number of reviews of utlrasound elastography and clinical guidelines, and they can be consulted to deepen knowledge in the technical and clinical domains [2,8,202,203,204,205,206].

There are several tests available in the clinical protocols to assess the extent of fibrosis and cirrhosis. The most common is a percutaneous liver biopsy, a procedure performed without hospital admission that consists of introducing a biopsy needle through the ribs to the liver [207]. Although it is a standardized method to determine the state of the liver, its limitations stem from being an invasive method, which can cause minor or severe complications [208]. Additionally, the liver is a large organ and the biopsy represents only 1 of 50,000 of its total volume, whereby it can provide false negatives or misinterpretations of the real state of the disease [209,210]. The METAVIR scale and the Scheuer classification [211] divides fibrosis into five stages. Stage 0: there is no fibrosis. Stage 1: mild fibrosis. Stage 2: fibrosis extends to areas near the portal vein. Stage 3: fibrosis extends out from the areas of the portal vein. In this stage, many bridges of fibrosis connect the portal vein with the central areas of the liver. Stage 4: fibrosis has evolved to cirrhosis, which is an advanced pathological stage with distortion of the hepatic vasculature and architecture [212].

The most important advance for fibrosis staging has been obtained with the appearance of transient elastography (TE) using Fibroscan^®^ (Echosens, Paris, France), which has pioneered efforts since its first commercialization in 2003. Fibroscan^®^ generates images corresponding to the propagated elastic wave associated with values of hepatic rigidity measured in kilopascals (kPa). In vivo results of Ziol et al. [213] and Castera et al. [214] indicate that TE allows differentiating significant states of fibrosis. Chon et al. in [215] confirmed in a meta-analysis that TE is more accurate for detecting F4 fibrosis than mild fibrosis. Similar results were obtained by Afdhal et al. [216]. Transient elastography has been shown to be effective in diagnosing cirrhosis (stage F4 fibrosis) and generally in distinguishing significant fibrosis (≥F2) from non-significant fibrosis (F0 and F1). Cassinoto et al. [217] made a comparison study between TE and 2D-SWE and pSWE using biopsy as a gold standard. Results demonstrate that shear wave elastography (SWE) is more accurate in the diagnosis of severe fibrosis than TE. Similar results can be found in [218,219,220]. However, the distinction between individual fibrosis stages is still not well validated. These studies did not change the frequency of vibration, thereby disregarding the viscoelastic properties of the liver, and this presumably could lead to errors in the early detection of liver fibrosis because the elasticity can be kept within normal values in those stages [110,220].

The highly viscoelastic structure of the liver suggests a strong diagnostic potential of viscosity, since shear wave velocity is frequency-dependent; this means that it is possible to in vivo quantify the tissue viscosity from the dispersion curves [99,122,221,222,223]. The elasticity of the liver depends mainly upon the fibrosis stage, but additionally on factors such as edema, inflammation, extrahepatic cholestasis and congestion [224]. For these cases of hepatic diseases, having an additional biomarker to quantify the stage of the disease may yield a significant advantage. Viscosity also plays a vital role in cases where the contrast of the elastography is not good enough [128].

In terms of attenuation of shear waves, viscosity has been used to propose a technique to separate transplanted livers with severe rejection from livers with no rejection by Nenadic et al. [105]. The study computed the attenuation of shear wave elastography (AMUSE), which allows the characterization of viscoelastic parameters without using rheological models. Shear wave velocity and attenuation of 15 transplanted livers in patients with severe rejection were measured; the results were correlated with biopsy findings, confirming a high ratio of concordance.

**Figure 5:**
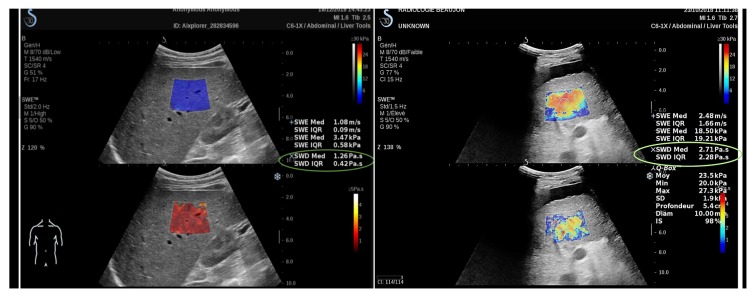
Measuring real-time viscosity of a volunteer patient using supersonic imagine (SSI) AIXPLORER MACH30^®^. The image on the left shows a healthy patient, while the right subfigure clearly distinguishes differences in the viscosity of a cirrhotic liver. Courtesy of Pr V.Vilgrain—Hopital Beaujon.

SSI was also used to staging liver fibrosis, with several studies reporting that shear wave imaging was more accurate than TE [225,226], but again, SWE can not reliably differentiate between mild stages of fibrosis. The importance of this potential biomarker has led to supersonic shear imaging (SSI) to recently release AIXPLORER MACH30^®^ (SuperSonic Imagine, Aix-en-Provence, France) with new liver tools as the viscosity imaging feature. Figure 5 shows and imaging of a healthy liver with real-time viscosity values.

Conversely, several authors obtained results that have shown that viscosity does not notably improve liver fibrosis staging [99,110]. The works of Chen et al. [98] and Lin et al. [100] used SDUV, reporting values of 1.96 ± 0.34 Pa·s for the in vivo healthy porcine liver and 1.07 ± 0.12 (F0), 1.22 ± 0.25 (F1), 1.61 ± 0.17 (F2), 1.64 ± 0.11 (F3) and 1.61 ± 0.21 Pa·s for the in vivo fibrotic rat liver, respectively. But the common understanding is that viscosity in the human liver increases with higher fibrosis stages, as summarized in Table 6. Likewise, a recent study of Sugimoto et al. [109] has tried to overcome the limitations of the former studies by enrolling subjects with a single etiology, and using the dispersion slope value instead of a simple Voigt model since there is no consensus in the clinical/elastography community with the most appropriate rheological model for soft tissue characterization. They put the focus of dispersion slope measurements on the lack of practical guidance. Furthermore, the work has confirmed that shear wave speed (SWS) is superior to shear wave dispersion slope in delimiting the degree of fibrosis. On the other hand, they found that the dispersion slope is superior to SWS in the prognostics of the degree of necroinflammation.

Moreover, it has been found that shear wave dispersion is strongly correlated with the degree of steatosis in non-alcoholic fatty liver (NAFLD). In the most severe cases NAFLD could progress to cirrhosis, requiring liver transplant [227]. Preliminary Ex vivo and in vivo studies in mouse, porcine, duck and goose livers manifest that viscosity may become a key biomarker in distinguishing fatty liver [128].

Recent publications have highlighted the interest MRE causes as a method for detection and staging of liver fibrosis. Sherman et al. [228] examined performance characteristics of the enhanced liver fibrosis (ELF) index compared to MRE. The conclusions stated that the ELF index was a highly sensitive and specific marker of cirrhosis when compared with MRE. A posterior study evaluated the relationship between an increase in liver stiffness on MRE and fibrosis progression in nonalcoholic fatty liver disease (NAFLD) [229]. The prospective cohort study included 102 patients who underwent contemporaneous MRE and liver biopsy. The study concluded that a 15% increase in liver stiffness on MRE may be associated with histological fibrosis progression. Although high mortality is associated with significant hepatic fibrosis, data on the estimated prevalence of liver fibrosis in the general population is scarce. Kang et al. [230] carried out a study with 2170 participants. The prevalence values of significant and advanced liver fibrosis were 5.1% and 1.3% in the overall health-clinic cohort.

Viscosity imaging seems to be an essential non-invasive biomarker, providing additional information to diffuse liver pathology. Even so, it is believed that suboptimal shear wave signal quality measured in vivo could be one of the causes of worse performance of viscosity over elasticity. The precise quantification of the viscosity is a challenging inquiry; besides, the selected viscoelastic model determines the accuracy of the results. Exploring the nonlinear parameters to evaluate the degree of fibrosis has not yet been achieved at any level.

### 3.4. Labor Disorders

The World Health Organization (WHO) estimated in 2017 that approximately 15 million babies would be born preterm (<37 weeks of gestation); this is a rate above 1 in 10 newborns [86]. The problem of cervical insufficiency is intimately related to the mechanical properties of the cervix, and hence any approach must involve means to quantify the biomechanical state of the cervix. The mechanical parameters are sensitive to the collagen remodeling that progresses throughout cervical ripening, and which ultimately controls the cervix’s mechanical ability to dilate [231].

Cervical tissue elasticity has been studied extensively. The first investigations were carried out by using static elastography (SE) [232]. However, researchers have claimed since then that we should not depend on SE to capture the changes that the cervical tissue undergoes during gestation because it highly depends on the pressure applied by the operator. Standardization of the measurement method is a call in many in vivo studies [233,234]. Molina et al. [235] came up with the idea of restricting the induced probe displacement. Controlling the pressure was an objective of Hernandez et al. [236], using a reference elastomer material [237]. Thus far it seems that there is no way to bypass the limitation of strain elastography [234,238,239].

Research moved towards looking for solutions, adopting the dynamic technique named shear wave elasticity imaging (SWEI) [51,240]. It has been widely used for the assessment of cervical changes [4,241,242,243,244,245]. Carlson et al. [4] measured SWS in human Ex vivo samples. Results showed that SWS was able to distinguish between ripened and unripened cervical tissue. Feltovich et al. [233] proposed the elasticity as an interesting biomarker for physicians, since the elastic modulus varies more than 80 kPa while SWS varies from approximately 1.2 to 5.5 m/s over the cervix. Carlson et al. [246] found in a longitudinal study that stiffness decreased over the course of pregnancy, and the same group explored the feasibility of SWS in capture the cervical softness in pre and post ripening in women experiencing induction for labor [241]. Peralta et al. [247] used the commercial SSI to quantify the cervical stiffness at four ROIs, which evidenced that microstructural changes generate a measurable shear stiffness reduction that gradually undergoes throughout gestation. This remodeling has been further investigated in the regions of the external os that have been proven to be softer than the internal os [235,248]. If pregnant women score small strain values at the internal os, it is unusual to experience spontaneous preterm birth [248]. SWS was found to decrease versus gestational age at the internal os [243]. Related results were obtained by Muller [242] in pregnant women compared to a control group. SWS before and after prostaglandin application were measured prior to term induction of labor in 20 women. Significant results were obtained (2.53 ± 0.75 m/s before and 1.54 ± 0.31 m/s 4 h after prostaglandin application) [241]. Authors also compared SWS between pregnant women in the first trimester and the third one; results of 4.42 ± 0.32 m/s and 2.13 ± 0.66 m/s were reported respectively.

Although the SWEI technique has been effective in the cervical tissue description, it presents some limitations: first, shear waves are highly attenuated due to the microstructural complexity of the cervix, and secondly, the complexity of producing adequate shear waves in its boundaries. The use of torsional waves (shear elastic waves that propagate radially and in-depth in a curved geometry to sense soft tissue architecture) has been demonstrated to enable a new class of characterization to quantify the mechanical functionality of any soft tissue [249,250,251,252].

Given these limitations, Melchor et al. [253] and Callejas et al. [111] introduced a novel technique, torsional wave elastography (TWE). The method is based on the transmission of shear waves by a rotational electromechanical actuator and received by a sensing ring. One of the advantages of this technique when compared with SWEI, is that it is highly adequate for cylindrical, small organs, such as the uterine cervix, since TWE generates low energy that does not generate rebounds as SWEI. Torsional wave elastography was used to quantify the stiffness of cervix in pregnant women in vivo by Masso et al. [254]. Preliminary results reveal that TWE could become an advantageous technique capable of quantifying the decrease of cervical stiffness during gestation.

**Figure 6:**
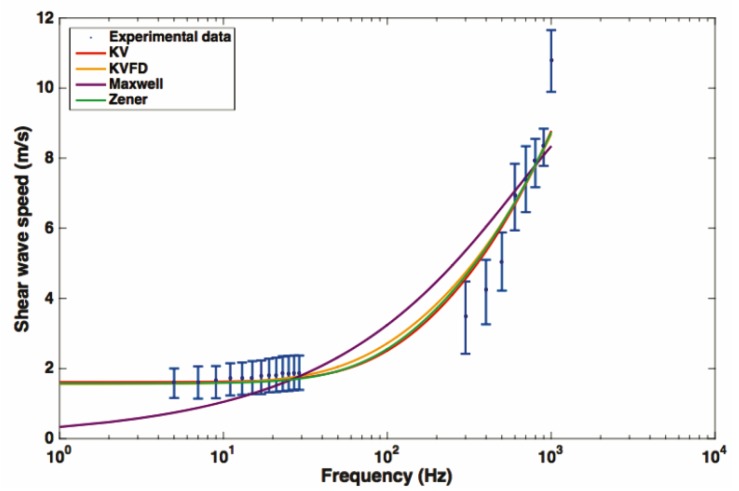
Fitting of the most popular rheological models to the experimental results obtained by rheometry (the lowest frequencies) and TWE (the highest frequencies) in the cervix Ex vivo. The Kelvin–Voigt (KV), Kelvin–Voigt fractional derivative (KVFD) and Zener models are successfully adjusted while the Maxwell model is not able to represent the full frequency range satisfactorily. Source: Sensors, reproduced from 2017 Callejas et al. [111].

Up to this point, the studies presented earlier have ignored the viscosity and nonlinearity of the uterine cervical tissue. Substantial hydration changes and inflammatory processes are well known to occur during maturation, as is collagen decrimping, which suggests that viscous and nonlinear parameters may be of significant importance. TWE explored viscosity in Ex vivo cervix tissue—results are shown in Table 7 and Figure 6 [111]—and nonlinear parameters by the harmonic generation of torsional shear waves [145].

McFarlin et al. [255] suggested that cervical ultrasonic attenuation, which is theoretically linked to compressional viscosity (independent from shear viscosity), could identify women at risk of spontaneous preterm birth (SPTB). It seemed that low attenuation may be an additional biomarker with which to identify SPTB. SWEI was conducted in vivo on the pregnant cervix of Rhesus macaque, divided into two groups; ripened and unripened specimens [81]. Authors found dispersion (the slope of dispersion curve of SWS versus frequency) in both groups (median 5.5 m/s/kHz, interquartile range: 1.5–12.0 m/s/kHz). Peralta et al. [71] proposed Maxwell’s model as the best model to use in preliminary estimations of cervical viscoelastic properties. Myers et al. [256] suggested that since the cervical tissue is mechanically anisotropic, the uniaxial response of Ex vivo human cervix samples would depend on the load direction.

Jiang et al. [257] employed 3D multifrequency MRE to the uterus and analyzed the viscoelasticity of the uterine tissue in healthy volunteers. They observed that the uterine corpus has higher elasticity, but similar viscosity compared with the cervix, in terms of complex shear modulus (uterine corpus = 2.58 ± 0.52 kPa vs. cervix = 2.00 ± 0.34 kPa). They concluded that the proposed technique shows sensitivity to structural and functional changes of the endometrium and myometrium during the menstrual cycle. Shi et al. [258] measured the compressive viscoelastic mechanical properties of Ex vivo human cervical tissue using indentation and an inverse finite element analysis, to conclude that the human cervix is nonlinear and the area of the internal os is stiffer than the external os.

No human in vivo measurements of cervical viscosity changes during gestation have yet been reported in the literature, and no measurements of nonlinear biomarkers have been published as far as we know.

## 4. Discussion

In perspective, the purpose of this review was to present ground and clinical evidence that goes a step beyond linear elasticity. Abnormalities in the viscosity and nonlinearity of soft tissues are intimately linked to a broad range of pathologies, including labor disorders, solid tumors, atherosclerosis, liver fibrosis and osteoarticular syndromes, just to name but a few. This suggests that it is crucial to rethink where we are in terms of soft tissue mechanics and how pathologies affect them, opening a timely opportunity of moving forward defining new mechanical biomarkers, enabling earlier, more specific and precise diagnostic and therapeutic decision making.

On the one hand, viscoelasticity, or more generally, tissue rheology or dynamic dispersion, is recognized from the physics of wave propagation as a compound expression of the rheological, poroelastic and microstructural scattering phenomena governed by the complex fibrous multiscale microstructure of the stroma, which mainly stems from the interaction of collagen and elastin with the viscous proteoglycans, which undergo characteristic changes during pathologies.

On the other hand, the significant hyperelasticity that soft tissues exhibit can manifest itself as quantifiable shear wave harmonic generation, and one of the main hypotheses about the pathology-mediated origin of nonlinearity changes is based on the crimping and crosslinking of tissue fibers. In the same manner that shear waves have recently been believed far more sensitive to tissue classification than standard compressional waves but are troublesome to quantify; some experimental observations may tangentially suggest nonlinear mechanical properties may be a key signature with which to quantify and classify and diagnose a range of soft tissue pathologies.

Only scarce clinical elastography measurements of viscous or nonlinear parameters have been reported for diagnostic purposes, despite the promising perspectives that both unveil from the underlying rationale and from Ex vivo or animal testings. For instance, within the field of labor disorders, despite the decrimping of fibers along gestation as well as the inflammatory process, it is suggested to be a strong diagnostic potential of those biomarkers. Nonetheless, no attempts to measure viscoelastic and nonlinear parameters using elastography as biomarkers have been reported in the literature, which opens a promising research field. Similarly, Ex vivo measurements together with non-elastographic data evidence strong correlations between viscosity and pathology in the liver and prostate, supporting promising clinical potential and opening future research prospects. Within the field of breast cancer, only one attempt using shear wave elastography for nonlinear measurements in vivo has been reported to date, combining elastography with a prestress that modifies the shear wave speed due to the Landau-type elastic nonlinearity, though it exhibited limited repeatability. Still, MRE delivers more extensive results with a clearly discriminant potential. Despite these preliminary experiences, linear and nonlinear elastography, possibly together, promise an improved sensitivity and specificity to characterize benign and malignant mammary lesions.

Regarding the limitations of these recent methodologies, it is difficult to describe them objectively, since it is not possible to compare studies and draw conclusions. Viscosity measurement with ultrasonic techniques is currently less extended than by MR techniques, but this shortcoming is only attributable to the immaturity of the ultrasonic technique; thus, barriers to its future potential are foreseen. The two origins of dispersion: viscosity and poroelasticity will probably remain indistinguishable in vivo, since their separation would require measurements at timescales too far away. Hence, a single biomarker will probably describe both. Evidence towards the potential of elastic nonlinearity biomarkers has been provided, whilst the technology is still too immature to state any potential limitations towards nonlinearity quantification.

The key open research questions involve a detailed formulation for the nonlinear and viscous components of the microstructure as the ideal procedure to understand the changes and functions in tissues that exhibit these behaviors. However, the diverse interactions between fluid components and fibers do not allow the validation of complete models, where the stored energy is considered individually for each component, ignoring physiological processes of mixed nature that should not be underestimated. In the specific case of viscosity, the industry has already taken its firsts steps to address it at the clinical level, and the challenge now is that commercial elastography techniques must converge on a common framework for the estimation of viscosity and accurate differentiation of disease states, not only regarding whether there is a pathological condition, but whether it is of malignant or benign nature. In particular, ways to enhance the dispersion biomarker applicability, by widening the interrogation frequency range, promise to enable not only storage and loss moduli, but also poroelastic and a range of viscoelastic models simultaneously. This would yield more profound understanding of tissue rheological ultrastructure and histology parameters, eventually allowing prediction of how disease processes change mechanical properties. As regards nonlinearity, it is a yet pending biomarker, an emerging concept where the still-modest clinical experiences such as breast cancer A parameter promise strong diagnostic potential once the technical issues are solved. Nonlinearity is, to our knowledge, still not available on commercial systems.

In conclusion, several front lines have been exposed, yet many other questions call for a response. How do soft tissue properties change in the case of anisotropy tissues? How about on a cellular scale in the presence of tumors? How will the ultrasound elastography industry develop techniques considering these biomarkers to adapt them to a real application? Quantitative answers to these questions would definitely improve many clinical protocols.

## Figures and Tables

**Table 1:** Qualitative overview of the work done on the description of the viscoelastic nature of selected soft tissues. The techniques that have achieved remarkable results are: shear wave dispersion ultrasound vibrometry (SDUV), dynamic mechanical analysis (DMA), magnetic resonance elastography (MRE), shear wave elastography (SWE) and torsional wave elastography (TWE). KVFD: Kelvin–Voigt fractional derivate; KV: Kelvin–Voigt.

Technique	Soft Tissue	Study Objective	Method	Reference
SDUV	Liver in vivo porcine	Regular characterization	Dispersion curve Voigt model	Chen et al. [98]
	Liver in vivo	Regular characterization	Dispersion curve Voigt model	Chen et al. [99]
	Liver in vitro rat	Fibrosis staging	Dispersion curve Voigt model	Lin et al. [100]
	Prostate in vitro	Regular characterization	Dispersion curve Voigt model	Mitri et al. [101]
	Breast in vivo	Malignant vs. Benign vs. Healthy state	Dispersion curve Voigt model	Kumar et al. [89]
DMA	Prostate in vitro	Healthy vs. Cancerous state	Dispersion curve KVFD model	Zhang et al. [102]
MRE	Breast in vivo	Malignant vs. Benign vs. Healthy state	Phase offset imaging reconstruction	Sinkus et al. [103]
	Breast in vivo	Malignant vs. Benign vs. Healthy state	Transversely isotropic model	Sinkus et al. [104]
	Liver in vivo	Transplant rejection	Attenuation Measuring Ultrasound Shearwave Elastography (AMUSE)	Nenadic et al. [105]
	Liver in vivo	Regular characterization	Dispersion curve Zener model	Klatt et al. [106]
	Liver in vivo	Fibrosis staging	Dispersion curve Zener model	Asbach et al. [107]
	Prostate in vivo	Prostate cancer vs. Benign prostatitis	Phase offset imaging reconstruction	Li et al. [108]
SWE	Liver in vivo	Fibrosis	Shear Wave Dispersion Slope	Sugimoto et al. [109]
	Liver in vivo	Healthy vs. Fibrosis staging	Shear Wave Spectroscopy	Deffieux et al. [110]
TWE	Cervix Ex vivo	Regular characterization	Dispersion curve KV and KVFD model	Callejas et al. [111]

**Table 2:** Comparison of the current methods that have been able to successfully estimate viscosity parameters using ultrasound elastography.

Method	Advantages	Disadvantages
Shear wave speed dispersion curve: estimation of vicosity parameters by fitting a rheological model	Most relevant and extended technique Considerable amount of previous work for different types of organs to compare with Depends on shear wave methods: noninvasive both internally and externally in contact with the soft tissue	No consensus on the most appropriate rheological model for soft tissue characterization Studies report values of viscosity for a specific rheological model (not comparable)
Shear Wave Dispersion Imaging	Dispersion slope value: physical quantity not based on a rheological model (model-free)	Integrated into commercial ultrasound systems not accessible for researchers (black box software)
Shear Wave Spectroscopy: new signal processing of the SSI data (Supersonic Shear Imaging)	Frequency-dependent measurement of the shear wave speed, quantitative and noninvasive	Limits its use to scans via SSI

**Table 3:** Summary of the current state of implementation of nonlinearity in the quantification of soft tissue mechanical properties.

Advantages	Disadvantages
New set of parameters to interpret biological and physiological disorders	Several proposed models to be chosen depending on the problem, pathology or tissue considered
Characterization of tissue microscale in terms of harmonics	Inhomogeneus measurements due to the nature of propagation in the tissue
Open questions that add a new branch in biomedical engineering	Mathematically intractable in exact terms

**Table 4:** Viscosity parameters derived from different methods, including a Kelvin–Voigt fractional derivative (KVFD) fitting using dynamic mechanical analysis (DMA), KV fitting on shear wave dispersion ultrasonic vibrometry (SDUV) and magnetic resonance elastography (MRE) results of prostatic tissue. Values are reported as means and standard deviations.

Tissue State	Viscosity Parameter (Pa.s)	Fractional Derivate Order	Method	Reference
Healthy	3.61 ± 1.25	0.215 ± 0.042	DMA	Zhang et al. [102]
Cancerous	8.65 ± 3.40	0.225 ± 0.03		
Healthy	1.10–6.82 (range)	-	SDUV	Mitri et al. [101]
Benign prostatitis	2.13 ± 0.21	-	MRE	Li et al. [108]
Cancerous	6.56 ± 0.99	-		

**Table 5:** Viscosity parameters are calculated for the malignant, benign and healthy states in the breast tissue. The methods applied were magnetic resonance elastography (MRE), transverse acoustic waves and shear wave dispersion ultrasound vibromerty (SDUV). Values are reported as means and standard deviations.

Tissue State	Viscosity Parameter (Pa.s)	Method	Reference
Malignant	2.40 ± 1.70	MRE	Sinkus et al. [103]
Benign	2.10 ± 1.40		
Healthy	0.55 ± 0.12		
Malignant	3.00 ± 0.80	Transverse Acoustic Waves	Sinkus et al. [104]
Benign	2.40 ± 1.90		
Healthy	0.70 ± 0.55		
Malignant	8.22 ± 3.36	SDUV + Kelvin-Voigt	Kumar et al. [89]
Benign	2.83 ± 1.47		
Healthy	1.41 ± 0.67		

**Table 6:** Human liver range of viscoelastic biomarkers for healthy state and different grades of fibrosis. Results were obtained using magnetic resonance elastography (MRE) and shear wave pectroscopy (SW spectroscopy). Values are reported as means and standard deviations.

Tissue State	Viscosity Parameter (Pa.s)	Method	Reference
Healthy	6.7 ± 1.3	MRE + Zener model	Klatt et al. [106]
Healthy	7.3 ± 2.3	MRE + Zener model	Asbach et al. [107]
Healthy	2.0 ± 0.8 (F0)	SW spectroscopy	Deffieux et al. [110]
	2.3 ± 0.7 (F1)		
Fibrosis	2.6 ± 0.5 (F2)	SW spectroscopy	Deffieux et al [110]
	2.7 ± 1.9 (F3)		
	3.7 ± 2.5 (F4)		
Fibrosis	14.4 ± 6.6 (F3–4)	MRE + Zener model	Asbach et al. [107]

**Table 7:** Viscoelastic parameters of Ex vivo cervical tissue using data from rheometry (R), torsional wave elastography (TWE) and a combination of both techniques (R + TWE) for Kelvin–Voigt (KV) and Kelvin–Voigt fractional derivative (KVFD) models. Values are reported as means and standard deviations.

Models	Rheometry (R)	TWE	R + TWE
Elasticity μ (kPa)			
KV	1.79 ± 0.08	2.43 ± 0.26	1.92 ± 0.15
KVFD	0.92 ± 0.15	2.06 ± 0.11	2.01 ± 0.24
Viscosity η (Pa.s)			
KV	6.34 ± 0.95	4.59 ± 0.29	4.5 ± 0.25
KVFD	23 ± 9.84	4.23 ± 0.22	4.64 ± 0.09
Fractional Derivative Power α			
KVFD	0.25 ± 0.15	0.97 ± 0.02	0.98 ± 0.01

## Abbreviations

The following abbreviations are used in this manuscript:

MRI	Magnetic Resonance Imaging
ECM	Extracellular Matrix
PGs	Proteoglycans
GAGs	Glycosaminoglycans
SMC	Smooth Muscle Cell
KV	Kelvin–Voigt
TOEC	Third-Order Elastic Constant
FOEC	Fourth-Order Elastic Constant
ARFI	Acoustic Radiation Force Impulse
pSWE	Point Shear Wave Elastography
TR-SWE	Transrectal Shear Wave Elastography
ROI	Region Of Interest
SDUV	Shear Wave Dispersion Ultrasound Vibrometry
KVFD	Kelvin-Voigt Fractional Derivative
DMA	Dynamic Mechanical Analysis
MRE	Magnetic Resonance Elastography
ELF	Enhanced Liver Fibrosis
WFUMB	World Federation of Ultrasound in Medicine and Biology
TE	Transient Elastography
SWE	Shear Wave Elastography
NAFLD	Non-alcoholic Fatty Liver Disease
SSI	Supersonic Shear Imaging
WHO	World Health Organization
SE	Static Elastography
SWEI	Shear Wave Elasticity Imaging
SWS	Shear Wave Speed
TWE	Torsional Wave Elastography
SPTB	Sponteneous Preterm Birth
